# Integrated Metabolome and Transcriptome Analyses Provides Insights into Ovule Abortion in *Camellia oleifera*

**DOI:** 10.3390/plants14040613

**Published:** 2025-02-18

**Authors:** Yayan Zhu, Jiajuan Xu, Gang Wang, Feng Xiao, Minggang Zhang, Qinmeng Zeng, Jie Xu

**Affiliations:** 1 Guizhou Academy of Forestry, Guiyang 550005, China; zyynjfu@163.com (Y.Z.);; 2 Institute for Forest Resources and Environment of Guizhou, Guizhou University, Guiyang 550025, China

**Keywords:** abortion, *Camellia oleifera*, LC-MS, ovule, abortion, transcriptomics

## Abstract

*Camellia oleifera* is a unique woody edible oil tree species in China, and the ovule development affects the yield of seeds. This study selected three different types of *C. oleifera* clones and used LC-MS, RNA-seq, and other techniques to compare the endogenous hormone contents, gene expression levels, and metabolite changes between normal and aborted ovules. The results showed that high levels of ABA, JA, and SA may lead to the phenotype of ovule abortion. A total of 270 differential metabolites were identified in the metabolome, with L-methionine, citrulline, L-tryptophan, L-phenylalanine, and indolepyruvate being downregulated to varying degrees in the aborted ovules. Genes involved in plant hormone synthesis and response, such as *GH3.1*, *IAA14*, *PIN1*, *AUX22*, *ARF1_2*, *BZR1_2*, *GA2ox*, *ERFC3*, *ABF2*, and *PYL8*, responded to ovule development. This study elucidates the physiological, metabolic, and transcriptional responses to ovule abortion, providing a theoretical basis for understanding ovule development and yield regulation in *C. oleifera*.

## 1. Introduction

*Camellia oleifera* (Fam.: Theaceae; Gen.: *Camellia*) is one of the most important woody oil species in China. Camellia seed oil is a high-quality woody oil, with an unsaturated fatty acid content as high as 80–90%, and its fatty acid composition is similar to that of olive oil, earning it the title of “Oriental Olive Oil” [1]. Additionally, Camellia oil contains abundant vitamins, sterols, squalene, polyphenols, camellia saponins, and other bioactive components [2], which have significant economic and medicinal value. The seed yield of *C. oleifera* is the primary economic trait, and the single fruit weight shows a highly significant positive correlation with the number of seeds [3]. Seed number is related to the number of ovules in the mature ovary and the ovule abortion rate. The number of ovules and the ovule abortion rate indirectly and synergistically affect the size of *C. oleifera* fruits by altering the ratio of fruits with different numbers of fertile locules [4]. *C. oleifera* fruits have three or four locules, containing more than 20 ovules. As the fruit develops, most of the ovules degenerate, leaving only one to six that eventually develop into mature and plump seeds [5]. Early in the development of *C. oleifera* fruits, there is an occurrence of hollowing in the ovules with abnormal embryo sac structures [6]. At 21 weeks after anthesis (WAA), the ovules are relatively uniform in size and white in color, making it difficult to distinguish between normal and aborted ovules. By 26 WAA, normal ovules begin to swell, creating a visible size difference between the two types. After 31 WAA, some ovules become sterile, while fertile ovules occupy most of the space within the ovary [7]. The proportion of aborted ovules varies among different varieties of *C. oleifera* plants, and they gradually shrivel and turn brown during later development stages [8]. During the rapid fruit development period, insufficient nutrient supply can lead to a large number of ovule abortions, which, in turn, can cause flower and fruit drop [9].

Ovule is an important reproductive organ and is the developmental precursor of seeds [10]. Ovule initiation determines the maximum number of ovules, thus significantly impacting the number of seeds per fruit and seed yield [11]. Ovule development is influenced by ribosome biogenesis, chromosome modification, substance and energy metabolism, plant hormone signal transduction, and cell cycle regulation, among others [12]. Auxin plays a crucial role in all aspects of plant growth and development. ANT is involved in regulating the formation of ovule primordia and the integuments, while HEN1 indirectly negatively regulates the expression of *ARF6* and *ARF8* in the auxin signaling pathway by controlling miR167, leading to the abnormal development of female gametophytes including ovules [13]. GAs have an important role in controlling ovule initiation and, hence, ovule number and also highlighted the DELLA proteins as a novel component in the gene regulatory network that governs this key developmental process in plants [14]. In loquat seeds, both IAA and SA contents are significantly higher in normal ovules than in aborted ones, while the contents of ACC and ABA are significantly lower in normal ovules compared to aborted ones [15]. In *Arabidopsis thaliana*, the loss-of-function mutant *epfl2-1* (At4G37810 and AtEPFL2) exhibits reduced density and number of ovule primordia [3]. Mitogen-activated protein kinase (MAPK) cascades, ethylene signaling pathway, and NAC transcription factor genes showed upregulated expression in abnormal ovules of *Xanthoceras sorbifolium* [16]. Ovule development is regulated by multiple factors, and the causes and mechanisms of abortion are complex and varied. Understanding the mechanisms and pathways regulating ovule number has become an important aspect of research in plant science.

Transcriptomics enables the detection of the overall transcriptional activity of any species at the single-nucleotide level, and metabolomics is the omics discipline most closely related to the phenotype. The joint analysis of transcriptomics and metabolomics both predicts changes in metabolites from the transcriptional perspective and verifies the outcomes of gene transcription from the metabolic viewpoint, thus facilitating cross-verification among omics studies [17]. This study investigates the influencing factors of ovule abortion in *C. oleifera* by measuring endogenous hormones and conducting transcriptomic and metabolomic analyses on normal and aborted ovules (NO and AO group) from three different types.

## 2. Results

### 2.1. Analysis of Untargeted Metabolomics

**Figure 1:**
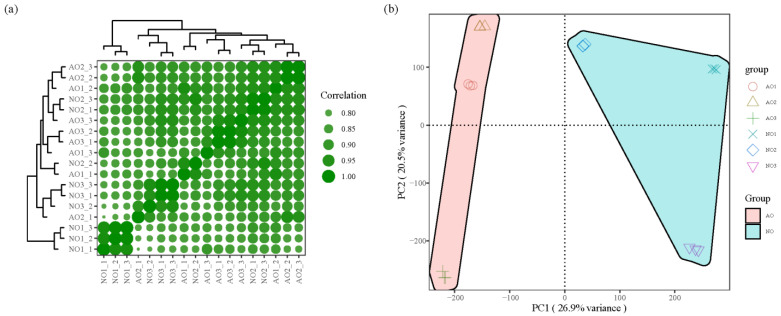
Correlation of samples and PCA of metabolomics. (**a**) Sample correlation analysis; (**b**) sample PCA. Note: In (**a**), the stronger the correlation, the larger the circle size. In (**b**), different samples were represented by different shapes, and two groups were filled with different colors.

Through the correlation analysis of samples, good reproducibility was observed among the samples. PCA revealed that PC1 and PC2 accounted for 26.9% and 20.5% of the total variance; there was a clear separation between the aborted ovules and normal ovules of the three types of *C. oleifera* (Figure 1). In the AO1 vs. NO1 group, 614 differential metabolites (77.04%) were upregulated, and 183 (22.96%) were downregulated; in the AO2 vs. NO2 group, 444 differential metabolites (65.01%) were upregulated, and 239 (34.99%) were downregulated. Venn diagram analysis of differential metabolites between different combinations revealed that there were 270 differential metabolites common to the aborted and normal ovules of the three types of *C. oleifera*. KEGG enrichment analysis of differential metabolites showed that 65 pathways were enriched, mainly including biosynthesis of various plant secondary metabolites (map00999), phenylpropanoid biosynthesis (map00940), biosynthesis of cofactors (map01240), flavonoid biosynthesis (map00944), and tryptophan metabolism (map00380) metabolic pathways. In the phenylpropanoid biosynthesis pathway, 4-coumaryl alcohol, L-phenylalanine, and coniferin were significantly downregulated in the aborted ovules, which is associated with abnormal cell wall development in aborted ovules.

### 2.2. Analysis of the Endogenous Hormone Content

**Figure 2:**
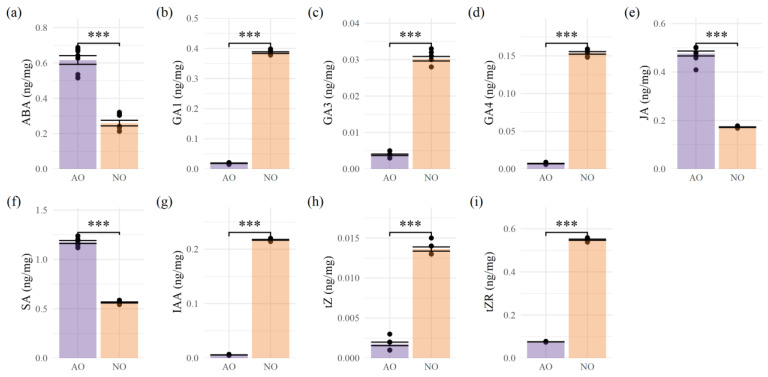
Changes in endogenous hormone content. (**a**): ABA hormone bar chart; (**b**): GA_1_ hormone bar chart;. (**c**): GA_3_ hormone bar chart; (**d**): GA_4_ hormone bar chart; (**e**): JA hormone bar chart; (**f**): SA hormone bar chart; (**g**): IAA hormone bar chart; (**h**): tZ hormone bar chart; (**i**): tZR hormone bar chart. The differences between the two groups were analyzed using *t*-test, where “***” indicated *p* < 0.001.

In aborted ovules, the contents of ABA, JA, and SA were significantly higher than those in normal ovules, with concentrations of 0.617 ng/mg, 0.476 ng/mg, and 1.177 ng/mg. In contrast, the contents of ABA, JA, and SA in normal ovules were 0.263 ng/mg, 0.173 ng/mg, and 0.565 ng/mg, respectively. Notably, the ABA content in aborted ovules was 2.346 times higher than that in normal ovules. Additionally, the contents of GA_1_, GA_3_, GA_4_, IAA, tZ, and tZR were all significantly lower in aborted ovules than in normal ovules. The IAA was 0.216 ng/mg in normal ovules, with a coefficient of variation (*CV*) of 1.446%, whereas in aborted ovules, it was only 0.06 ng/mg, with a *CV* of 9.686%; the tZR content in normal ovules was 0.550 ng/mg, with a *CV* of 1.314% while it was 0.076 ng/mg in aborted ovules, with a *CV* of 1.825%. There were extremely significant differences in the hormone contents between the two groups (Figure 2).

### 2.3. Analysis of Transcriptome Results

**Figure 3:**
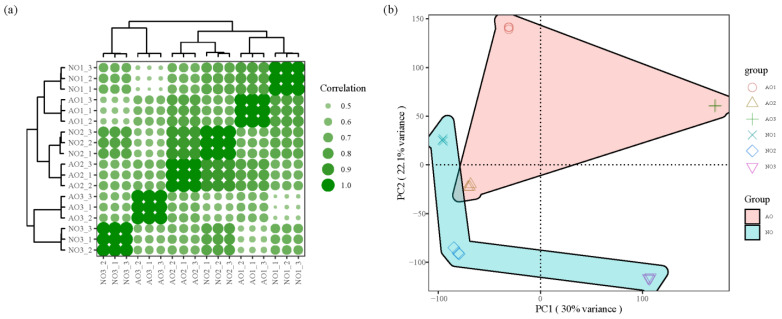
Correlation of samples and PCA of transcriptomes. (**a**) Sample correlation analysis; (**b**) sample PCA; Note: In (**a**), the stronger the correlation, the larger the circle size. In (**b**), different samples were represented by different shapes, and two groups were filled with different colors.

The transcriptomes of samples were analyzed, with each sample’s clean data reaching over 5.98 Gb, Q20 bases above 98.53%, Q30 bases above 95.35%, and GC values ranging from 43.57% to 44.12%, indicating a consistent distribution of GC content across samples. Additionally, the PCC reported values of ≥0.99 between biological replicates. PCA of the samples revealed that PCA1 and PCA2 accounted for 30% and 22.1% of the variance, respectively (Figure 3), and there was a clear distinction between aborted ovules and normal ovules. These results indicate overall good sequencing quality and reliable data, suitable for further analysis.

In the AO1 vs. NO1 group, there were a total of 6895 DEGs, with 3934 genes upregulated and 2851 genes downregulated; in the AO2 vs. NO2 group, 1217 genes were upregulated and 1170 genes were downregulated; in the AO3 vs. NO3 group, 3321 genes were upregulated and 3328 genes were downregulated. Venn diagram analysis of DEGs between different combinations revealed that there were 1409 common DEGs among the three groups.

GO enrichment analysis was performed on the DEGs. In the molecular function (MF) category, the most enriched genes were associated with DNA-binding transcription factor activity (GO:0003700), transcription regulator activity (GO:0140110), protein heterodimerization activity (GO:0046982), and sequence-specific DNA binding (GO:0043565). In the cellular component (CC) category, pathways such as nucleosome (GO:0000786), DNA packaging complex (GO:0044815), and cell wall (GO:0005618) were enriched. In the cell wall (GO:0005618) category, genes such as *At4g14310*, *GL34*, and *Glyco_hydro_28* were significantly downregulated in aborted ovules.

**Figure 4:**
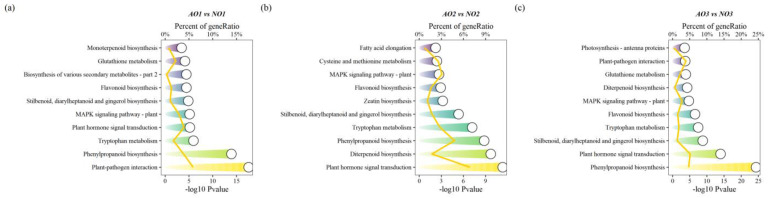
KEGG enrichment of DEGs. (**a**–**c**) were KEGG enrichment analysis plots for AO1 vs. NO1, AO2 vs. NO2, and AO3 vs. NO3, respectively. Note: The circles represented the −log_10_Pvalue of enriched terms, with different terms mapped in different colors. The line plot corresponded to the gene ratio values within each term.

KEGG enrichment analysis was performed on the DEGs (Figure 4), with a total of 575 DEGs annotated to KEGG pathways. Among these, 157 genes were significantly enriched in plant hormone signal transduction (map04075), diterpenoid biosynthesis (map00904), phenylpropanoid biosynthesis (map00940), tryptophan metabolism (map00380), and MAPK signaling pathway—plant (map04016). In the plant hormone signal transduction (map04075) pathway, genes involved in auxin response proteins, such as *GH3.1* and *GH3.9*, and the BR signaling transcription factor *BZR1_2*, were significantly downregulated. Ethylene response factor *ERFC3*, and genes related to ABA synthesis, such as *ABF2* and *PYL8*, were significantly upregulated in aborted ovules.

### 2.4. Transcriptomics and Metabolomics Integrated Analysis

A Venn diagram analysis of KEGG annotations shows that the gene set and transcript set are commonly annotated to 58 pathways, including plant hormone signal transduction (map04075), phenylpropanoid biosynthesis (map00940), etc., among which, tryptophan metabolism (map00380), zeatin biosynthesis (map00908), and other pathways are enriched.

Through the measurement of endogenous hormone content, it was found that, in aborted ovules, the levels of IAA, JA, and SA were significantly higher than in normal ovules, while GA_1_, GA_3_, GA_4_, IAA, tZ, and tZR were significantly lower than in normal ovules. In the metabolome, the contents of Gibberellin A32, Gibberellin A40, Gibberellin A7, multiple plant hormones, and precursors of secondary metabolites, such as Indolepyruvate, as well as key intermediates in auxin synthesis like 2-(1H-indol-3-yl)acetaldehyde, were decreased. Additionally, based on the results of transcriptome sequencing, genes related to the gibberellin catabolic process (GO:0045487), such as *GA2ox1*, *GA2ox2*, and *TIFY 9* from the JAZ family, were significantly upregulated; the salicylic acid-mediated gene *NPR1* was also upregulated; meanwhile, genes associated with auxin synthesis, including *IAA14*, *PIN1*, *PIN6*, *PIN1D*, *LAX5*, *AUX22D*, *AUX22*, *ARF1_2*, and *ARF5*, were significantly downregulated. In summary, the upregulation of genes related to GA catabolism, ABA, JA, SA, and ETH, along with the downregulation of genes related to IAA and CTK, led to changes in metabolites, thereby affecting the development of the ovules.

**Figure 5:**
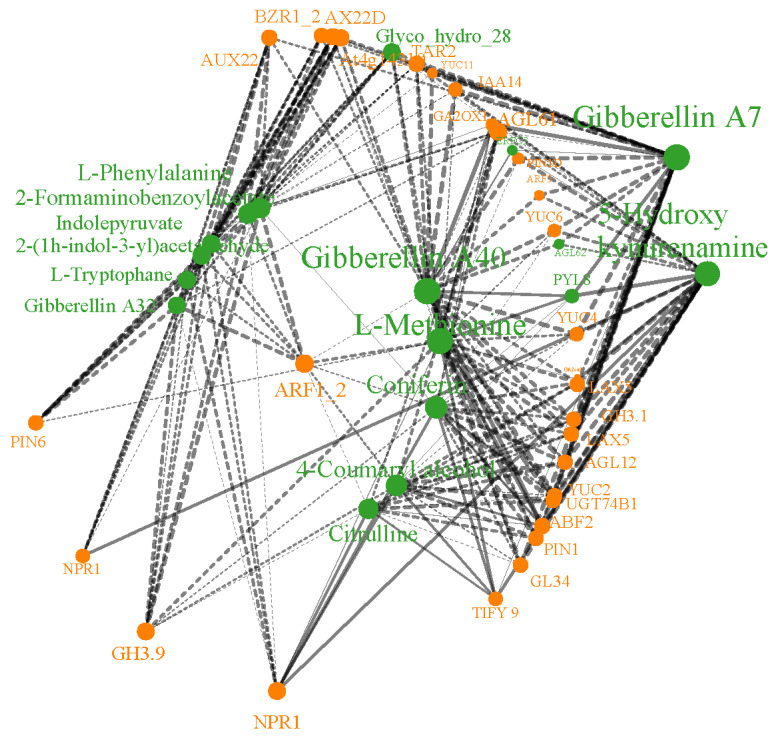
Network diagram of metabolites and gene correlations. Note: The layout used is focused. Metabolites are colored with #33a02c and genes with #ff7f00. Solid lines indicate positive correlations, while dashed lines indicate negative correlations. Node sizes reflect the strength of the correlations.

In the amino acid biosynthesis pathway, L-methionine, citrulline, L-tryptophan, and L-phenylalanine were significantly downregulated in aborted ovules. Tryptophan is one of the essential amino acids required for protein synthesis in plants and serves as a precursor for many important secondary metabolites. The KEGG enrichment analysis combining transcriptomics and metabolomics association showed that, in the tryptophan metabolism pathway, Indolepyruvate, 2-(1H-indol-3-yl)acetaldehyde, L-tryptophan, 5-hydroxykynurenine, and 2-formamidinobenzoylacetate were downregulated to varying degrees in aborted ovules. The downregulation of these metabolites is strongly correlated with the downregulation of YUCCA family members, *YUC2*, *YUC4*, *YUC6*, and *YUC11*, as well as UDP-glucose glycosyltransferase *UGT74B1* and the tryptophan aminotransferase encoding gene *TAR2* in the aborted ovules (Figure 5).

### 2.5. RT-qPCR

To verify reliability, eight genes related to the ovule development were selected from the transcriptome data for qRT-PCR. The results showed that the expression trends of these eight genes were basically consistent between the transcriptome data and the qRT-PCR results, indicating that the RNA-seq results have high reliability (Figure S1).

## 3. Discussion

The initiation of ovule primordia not only determines the maximum potential number of ovules per flower but also significantly influences the seed number per fruit and seed yield [18]. Among these factors, plant hormones play a central role, followed by transcription factors, enzymes, and other proteins, where AUX, BR, and CK act as positive regulators of ovule number, while GA acts as a negative regulator. Receptor proteins and small RNAs also participate in the regulation of ovule number by interacting with plant hormones and transcription factors [19]. During ovule development, auxin plays a crucial role in ovule initiation, and other hormones, such as CK [20], BR [21], and GA [14], also participate in regulating ovule formation and contribute to the size of the pistil and the number of ovules. These hormones can function independently or through cross-talk to influence ovule development [22,23]. In tetraploid locust trees, the levels of growth-promoting endogenous hormones (GA_3_ and IAA) in degenerated ovules are lower than those in normal ovules, while the level of growth-inhibiting endogenous hormone (ABA) and the ABA/(IAA + GA_3_) ratio are significantly higher than in normal ovules [24]. Compared with developing ovules, the levels of IAA, GA, CTK, and SA were found to be significantly lower in abortive ovules, while levels of ABA, ETH, and JA were found to be significantly higher in hazelnut [25]. The measurement of endogenous hormone levels in the normal and aborted ovules of *C. oleifera* showed that the hormone changes were basically consistent with those observed in hazelnut ovules. Furthermore, studies have found that exogenous IAA regulates the expression of genes related to flowering, ovule development, and fruit development, thereby affecting the fruit set rate [26].

With the development of sequencing technology, integrative analysis based on multi-omics has been widely proven to be an effective method for elucidating the biological characteristics of plants and the regulatory mechanisms of environmental responses [27]. Metabolomic and transcriptomic analyses were conducted on aborted ovules and normal ovules. KEGG enrichment results showed the following: SCMs were enriched in various pathways of plant secondary metabolite biosynthesis and tryptophan metabolism; DEGs were enriched in plant hormone signal transduction and zeatin biosynthesis pathways. In the metabolome, gibberellins, multiple plant hormones, and precursors of secondary metabolites such as Indolepyruvate and key intermediates in auxin synthesis like 2-(1H-indol-3-yl)acetaldehyde decreased. In the transcriptome, genes related to gibberellin degradation such as *GA2ox1*, *GA2ox2*, and *TIFY 9* of the JAZ family were significantly upregulated, as well as the ethylene response factor *ERFC3*, salicylic acid-mediated related gene *NPR1*, and ABA synthesis-related genes *ABF2* and *PYL8* in aborted ovules. Genes related to auxin synthesis, such as *GH3.1*, *IAA14*, *PIN1*, *AUX22*, and *ARF1_2*, and BR signaling transcription factors *BZR1_2* were significantly downregulated. Hormones such as auxins and cytokinins participate in regulating the complex molecular network of plant organ coordinated development. *ARF* and *AUX/IAA* auxin signaling genes have been identified as early auxin response genes [28]. Cytokinins regulate ovule development by modulating *PIN1* [29]. Additionally, auxin response factor *ARF2/MNT* may play a significant role in ovule development [30]. *GH3* encodes an auxin amide synthetase that catalyzes the conjugation of auxin with small molecule substrates containing acyl groups (such as amino acids and jasmonic acid), regulating plant growth and stress through modulating auxin homeostasis [31]. Arabidopsis lines overexpressing longan *DlGH3.5* and *DlGH3.6* exhibited phenotypes of stunted plants, fewer bolts, reduced flowering, shorter and fewer pods, and poor flower development [32]. Gibberellins are involved in critical developmental processes throughout the plant life cycle, from seed germination to stem and root elongation, flowering time, and fruit development [33,34]. DELLA proteins are a group of plant-specific transcriptional regulators, GRAS proteins, playing a crucial role in gibberellin signaling. DELLA proteins are new participants in determining the number of ovules in species such as *Arabidopsis* and agronomic crops like tomatoes and rapeseed, with their activity positively correlated with the number of ovules [14]. One of the DELLA proteins, *RGL2*, has also been confirmed to control flower development, ovule number, and fertility in Arabidopsis [35]. In the case of aborted ovules in *C. oleifera*, genes related to auxin synthesis such as *IAA14*, *PIN1*, *PIN6*, *PIN1D*, *LAX5*, *AUX22D*, *AUX22*, *GH3.1*, *GH3.9*, *ARF1_2*, and *ARF5* were significantly downregulated.

In the tryptophan metabolic pathway, Indolepyruvate, 2-(1H-indol-3-yl)acetaldehyde, and L-tryptophan were downregulated to varying degrees in aborted ovules. Genetic and biochemical studies have found that tryptophan is the main precursor for the synthesis of IAA [36,37]. The tryptophan-dependent IAA synthesis pathway mainly includes four types: (1) the indole-3-pyruvate (IPA) pathway; (2) the tryptamine (TAM) pathway; (3) the indole-3-acetamide (IAM) pathway; (4) the indole-3-acetaldoxime (IAOx) pathway [38,39]. Tryptophan-derived IPA is converted into IAA under the catalysis of YUCCA (YUC), which is also the rate-limiting step in auxin biosynthesis [40]. Studies have shown that the auxin synthesis pathway involving YUC plays a necessary regulatory role in developmental processes such as embryogenesis, flower organ development, and seedling growth in Arabidopsis [41]. In the tryptophan metabolic pathway, YUCC family members *YUC2*, *YUC4*, *YUC6*, and *YUC11* were significantly downregulated in the abortive ovules of *C. oleifera*, indicating that these genes are involved in the regulation of ovule development. Subsequent studies will involve genetic transformation of the *YUC2*, *YUC4*, and *YUC11* genes within the tryptophan metabolic pathway.

MADS-box genes play a role in almost every aspect of plant growth and development, including root development, flower development and evolution, hormone signal transduction, and fruit ripening [42]. The *AGL6* gene is involved in the regulation of floral meristems, flower organs, integuments, and seed development, and it may also play a role in male and female germ line and gametophyte development [43]. In Arabidopsis, *AGL9* and *AGL15* recruit the FIS-PRC2 complex to trigger the phase transition from endosperm proliferation to embryo development [44]. In defective ovules, the expressions of *AGL61*, *AGL62*, and *AGL12* were significantly downregulated.

## 4. Materials and Methods

### 4.1. Materials

The experimental materials were collected from a plantation in Liping County, Guizhou Province, which is one of the main production areas for *C. oleifera* in Guizhou. In late May (approximately 33 weeks after anthesis), three different types of 10-year-old clonal plants were selected. The sampling trees were naturally pollinated. At 9:00 AM, young fruits from different parts of the tree canopy were chosen, combined from a single plant, and the ovules were manually stripped and quickly frozen in liquid nitrogen, then stored at −80 °C for later use. This process was biologically repeated three times.

### 4.2. Untargeted Metabolomics Determination and Analysis

Based on the above samples, 100 mg of sample was taken into a 2 mL centrifuge tube and extracted with 800 µL of extraction solution (methanol–water = 4:1 (*v*:*v*)) containing four internal standards (L-2-chlorophenylalanine (0.02 mg/mL), etc.). The sample solution was ground for 6 min in a cryogenic tissue grinder at −10 °C and 50 Hz, followed by low-temperature ultrasonic extraction for 30 min at 5 °C and 40 kHz. The samples were then placed at −20 °C for 30 min and centrifuged 15 min (4 °C, 13,000× *g*). The supernatant was transferred for instrument analysis. Equal volumes of all sample metabolites were mixed to prepare quality control (QC) samples. LC-MS analysis was performed using a UHPLC-Q Exactive system (Shanghai Megi Biotech Co., Ltd.,Shanghai, China). Chromatographic conditions: 3 µL of the sample was separated on a BEH C18 column (100 mm × 2.1 mm i.d., 1.7 µm) before entering the mass spectrometer. Mobile phase A was 2% acetonitrile in water (containing 0.1% formic acid), and mobile phase B was acetonitrile (containing 0.1% formic acid). Raw data were processed using Progenesis QI (Waters Corporation, Milford, CT, USA), and principal component analysis (PCA) was performed using the ropls package (Version 1.6.2). Metabolites with VIP > 1, *p* < 0.05, and FC (fold change) > 1 or FC < 1 based on the OPLS-DA model were considered significantly differential metabolites.

### 4.3. Measurement and Analysis of Endogenous Hormones

Based on the above samples, 100 mg of each sample was weighed and placed into a 2 mL grinding tube. The target compounds in the samples were qualitatively and quantitatively analyzed using LC-ESI-MS/MS (UHPLC-Qtrap) with the following parameters: Chromatographic Conditions: Waters BEH C18 column (2.1 mm × 100 mm, 1.7 um), column temperature of 30 °C, injection volume of 10 µL; Mobile Phase A: 0.1% formic acid in water; Mobile Phase B: 0.1% formic acid in acetonitrile. The equilibration time is 3 min, and run time is 10 min. A linear regression standard curve was plotted with the mass spectrometry peak area of the analyte on the *y*-axis and the concentration of the analyte on the *x*-axis. The mass spectrometry peak area of the analyte in the samples was substituted into the linear equation to calculate the sample concentration results.

### 4.4. Library Construction and Transcriptome Analysis

Total RNA was extracted from the samples using Trizol, and RNA integrity was checked by electrophoresis. Concentration and purity were detected using NanoDrop 2000 UV-vis spectrophotometer (Thermo Scientific, Waltham, MA, USA) and the RIN value was measured using an Agilent 2100 (Agilent Technologies, Santa Clara, CA, USA). Library construction was performed using the Illumina^®^ Stranded mRNA Prep, Ligation (standard amount) method. The cDNA libraries were sequenced on the NovaSeq X Plus platform. The raw data were quality controlled using the fastp v0.20.1 software [45], and the clean data obtained after quality control were aligned to the reference genome using HiSat2 v2.2.1 to obtain annotation information [46]. Gene expression levels (TPM) were quantitatively analyzed using the RSEM v1.3.3 software [47]. Differential expression genes (DEGs) were identified using DESeq2 [48], with screening criteria set at |log_2_FC| > 2 and *p*-adjust < 0.05.

### 4.5. Metabolome and Transcriptome Correlation Analysis

Using the MegiCloud platform, calculate the Pearson correlation coefficient between SCMs and DEGs. A PCC closer to 1 indicates a higher degree of similarity in expression between genes and metabolites in the samples. The enriched pathways shared by both transcriptomic and metabolomic data were statistically analyzed. The ggnet R package was used to plot the interaction network diagram of metabolites and genes.

### 4.6. Quantitative Real-Time PCR (qRT-PCR) Analysis

Eight differentially expressed genes were selected, and RT-qPCR was performed using *GAPDH* as the internal reference gene to verify the reliability of the transcriptome data. PCR primers were designed using Oligo 7 (Table S1). The PCR conditions were as follows: preheating at 95 °C for 30 s, 40 cycles of heat denaturation at 95 °C for 5 s, and annealing at 58 °C for 34 s. The gene relative expression levels were calculated using the 2^−ΔΔCT^ method.

## 5. Conclusions

The yield is an important economic indicator, and ovule abortion affects the yield of *C. oleifera.* Through the determination of endogenous hormones, combined transcriptomic and metabolomic analysis, the results showed the following: in aborted ovules, the contents of ABA, JA, and SA were all significantly higher, while the contents of GA_1_, GA_3_, GA_4_, IAA, tZ, and tZR were all significantly lower than in normal ovules, indicating that abnormal endogenous hormones lead to ovule abortion. In the metabolome, L-methionine, citrulline, L-tryptophan, L-phenylalanine, 4-Coumaryl alcohol, 2-(1H-indol-3-yl)acetaldehyde, and Indolepyruvate were downregulated to varying degrees in aborted ovules. The transcriptomic results indicated that genes such as *GH3.1*, *IAA14*, *PIN1*, *AUX22D*, *ARF1_2*, and *BZR1_2* were significantly downregulated in aborted ovules, while genes such as *GA2ox*, *ERFC3*, *ABF2*, and *PYL8* were significantly upregulated in aborted ovules.

## Data Availability

The raw sequence data reported in this paper have been deposited in the Genome Sequence Archive in National Genomics Data Center, China National Center for Bioinformation/Beijing Institute of Genomics, Chinese Academy of Sciences (GSA: CRA020398) that are publicly accessible at https://ngdc.cncb.ac.cn/gsa, (accessed on 13 November 2024).

## Supplementary Materials

The following supporting information can be downloaded at: https://www.mdpi.com/article/10.3390/plants14040613/s1. Figure S1: qRT-PCR validation; Table S1: The primer list of qRT-PCR.

## Abbreviations

*C. oleifera*	*Camellia oleifera*
IAA	indole-3-acetic acid
GA	gibberellic acid
ABA	abscisic acid
tZ	trans-Zeatin
tZR	trans-zeatin-riboside
JA	jasmonic acid
SA	salicylic acid
GO	gene ontology
DEGs	differentially expressed genes
KEGG	The Kyoto Encyclopedia of Genes and Genomes
MADS-box	MADS-box gene family
TPM	transcripts per million
PCC	Pearson correlation coefficient
