# Supplementary material for: Integrated Metabolome and Transcriptome Analyses Provides Insights into Ovule Abortion in Camellia oleifera
Source: Plants (Basel). 2025 Feb 18;14(4):613. doi: 10.3390/plants14040613 (PMC11859457; doi:10.3390/plants14040613)
Supplement: Supplementary file 1 [file plants-14-00613-s001.zip › plants-3382712-supplementary.pdf]

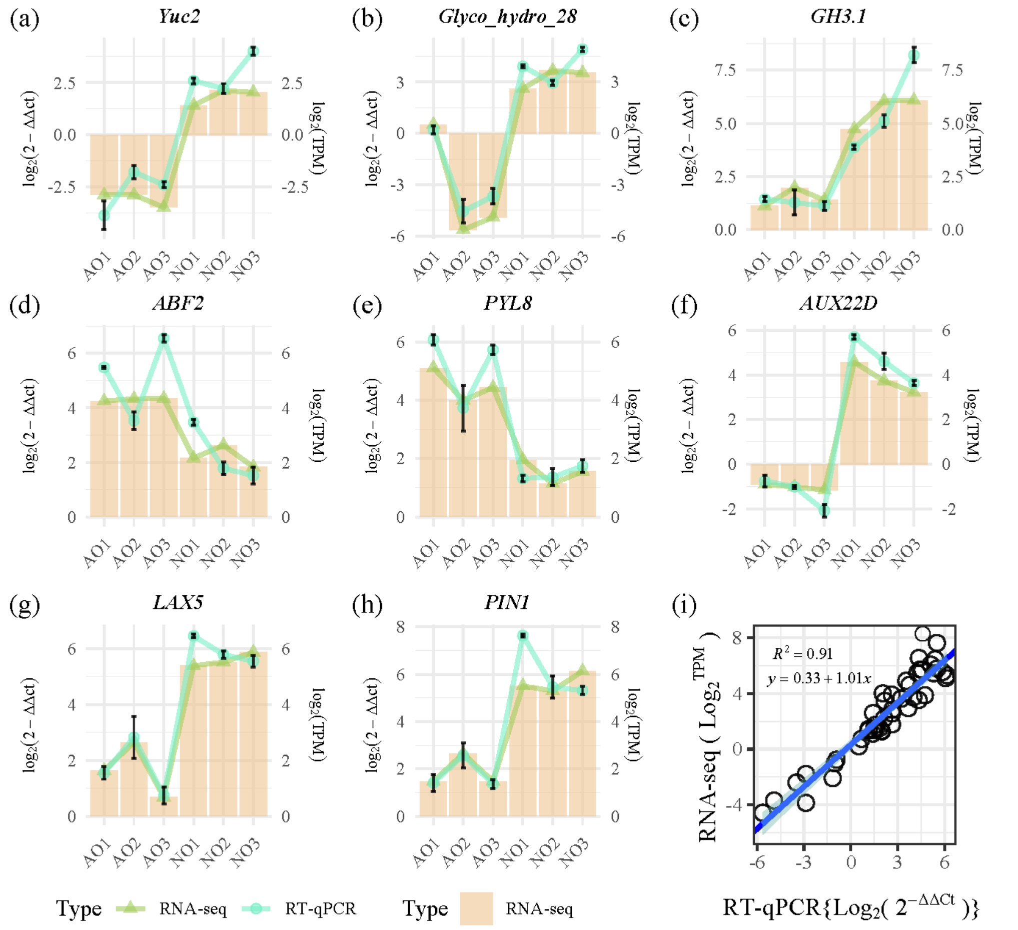

Figure S1. qRT-PCR validation.

Table S1. Primer sequences.

Gene name	Forward primer	Reverse primer
Glyco_hydro_28	CTGGCGGAAGGTTCATCAGT	TGCCAGGAAGGAGAGTTGAT
GH3.1	AGCAAGTCCTCCGTGTGGGT	CCTCCCATTTCTCATCTTCACA
ABF2	AGTGGTGTTGGAGAAGGGAA	CTCCTCCAGTGTCATCTCTC
PYL8	GGAAGCACCACAAGCACCAG	TCTTTCGGTGCTGGTAGTGG
AUX22D	AGATGATGGATGCTGCCCTG	CATCCCTGAACCATCTCCCT
LAX5	ATAAGGTGGTGGAGACTGTGAT	GAGCCACCTGATTTGAAGCACA
PIN1	CTTCGTTGCCCTCTTCGCTGT	TCTCTTGCTCACCTTACTCCAC
YUC2	CTTCAGCCTTCACCTTCCTA	CGCCTCCATTGTCAAACTCT
